# Supplementary material for: Noncoding de novo mutations in SCN2A are associated with autism spectrum disorders
Source: iScience. 2025 Aug 5;28(9):113258. doi: 10.1016/j.isci.2025.113258 (PMC12392654; doi:10.1016/j.isci.2025.113258)
Supplement: Document S1. Figures S1–S5 [file mmc1.pdf]

iScience, Volume *28*

Supplemental information

Noncoding *de novo* mutations in *SCN2A* are associated with autism spectrum disorders

Yuan Zhang, Mian Umair Ahsan, and Kai Wang

Supplementary Information:

Fig S1. Genetically inferred race/ethnicity by multidimensional scaling (MDS) analysis in SPARK

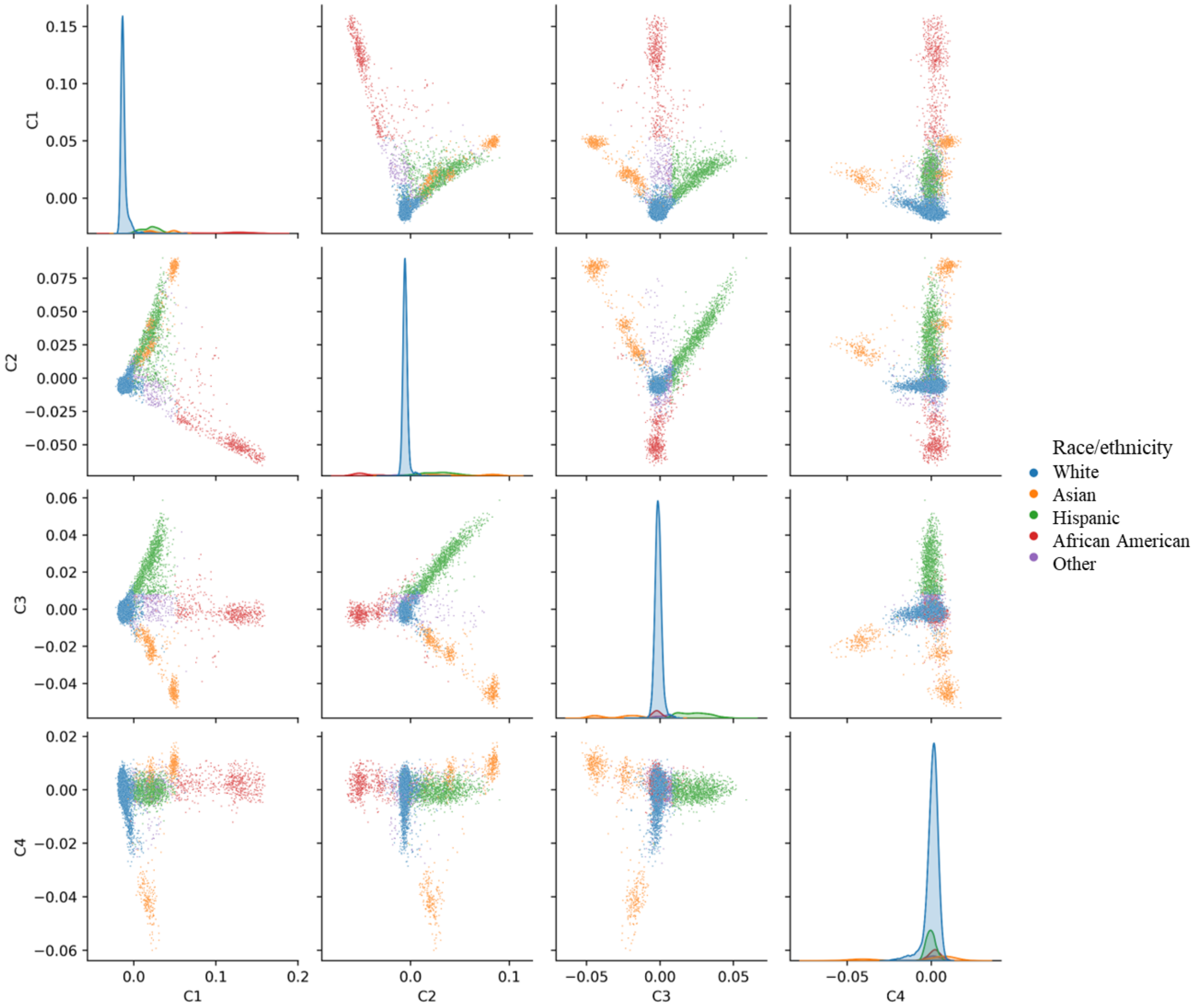

Fig S2. Distribution of DNMs in SSC. (a) distribution of coding DNMs. (b) distribution of missense DNMs. (c) distribution of loss of function DNMs. (d) distribution of noncoding DNMs. (e) distribution of generic noncoding DNMs. (f) distribution of intergenic noncoding DNMs. (g) distribution of noncoding DNMs have a CADD score ≥15. (h) distribution of generic noncoding DNMs have a CADD score ≥15. (i) distribution of intergenic noncoding DNMs have a CADD score ≥15.

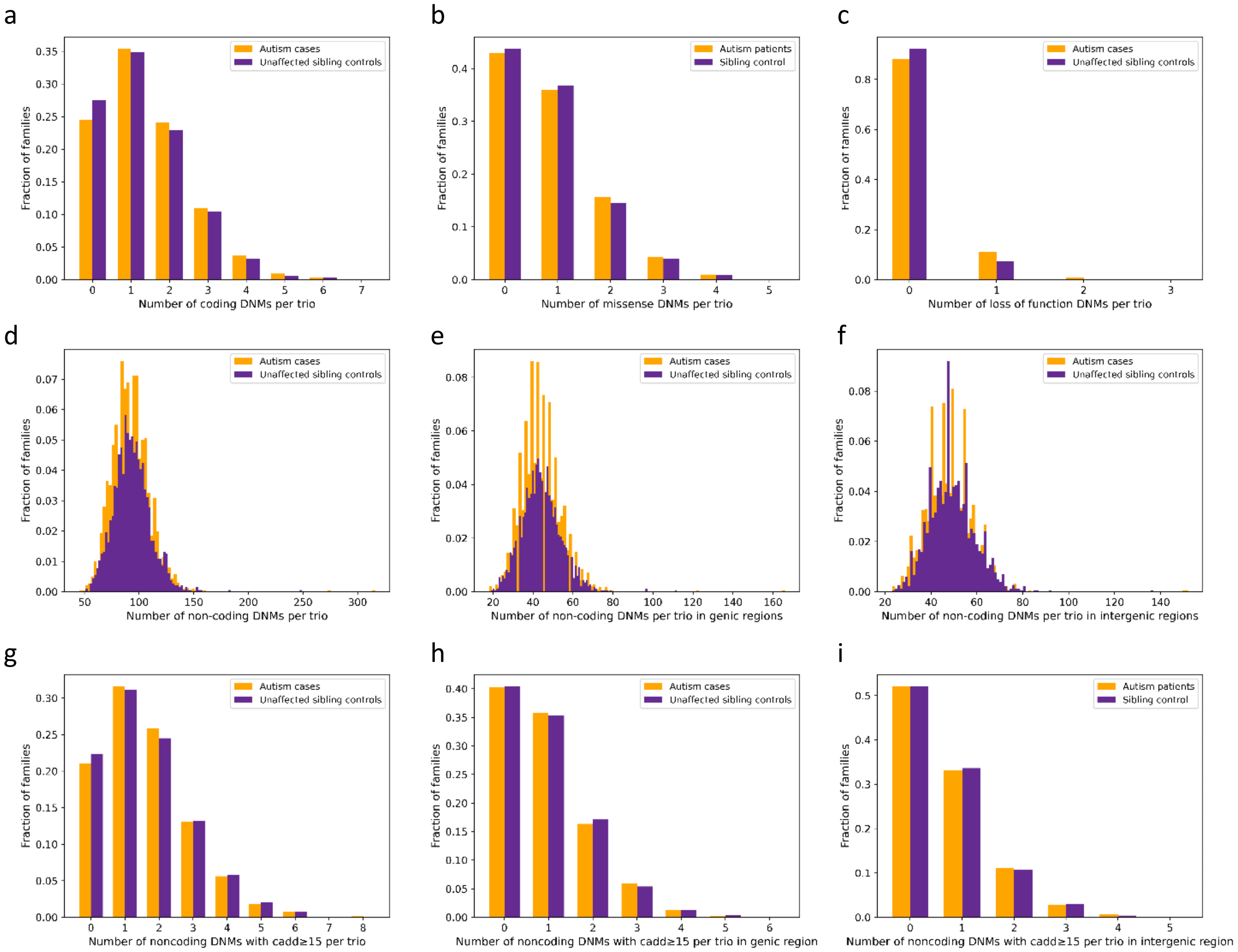

Fig S3. A category-wide association study (CWAS) analysis from SPARK cohort. (a) Burden test. Each dot in the plot is a category. The y axis refers to two-sided binomial p-values in -$\log_{10}$ format. The x axis refers to the relative risk in $\log_2$ format. The dashed line represents a p-value threshold of 0.05. (b) Detecting association with networks (DAWN) analysis for transcription factor binding sites (TFBS). The network illustrates the relationship of clusters, with node indicating the degree of disease association (z-score scale) and node size reflecting cluster size (number of categories).

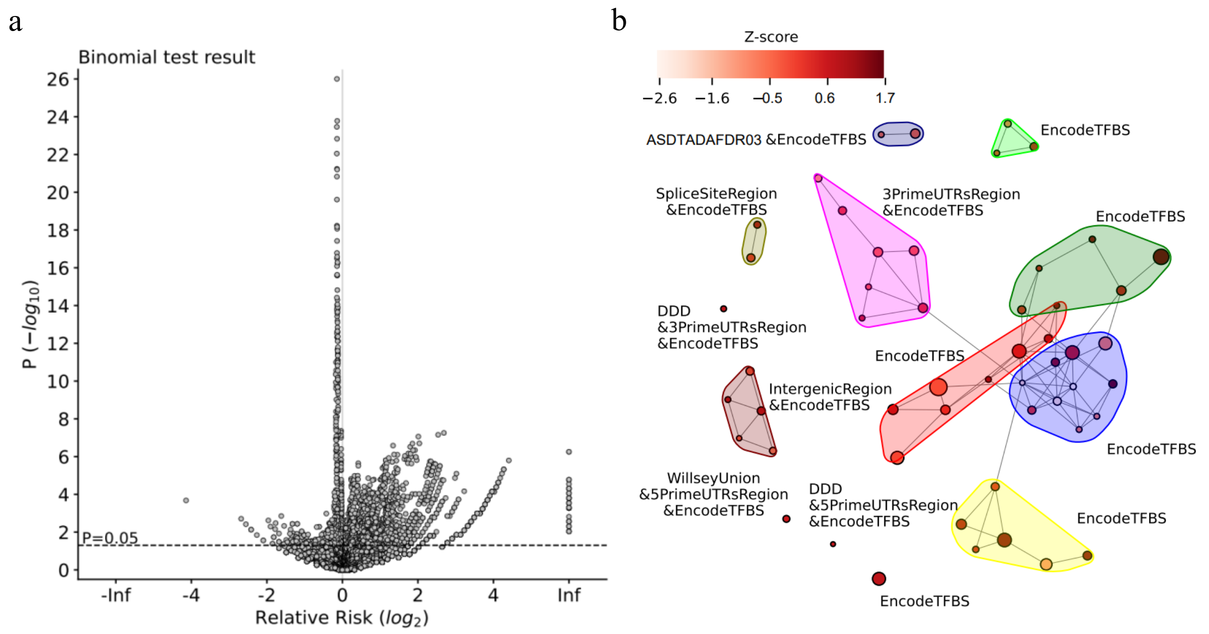

Fig S4. Mutation of the noncoding DNMs for *ZEB2* in SPARK. (a) mutation of chr2:144394497 (A>G). (b) mutation of chr2:144396373 (G>C). (c) chr2:144400613 (A>G). (d) mutation of chr2:144404388 (T>C). (e) mutation of chr2:144426509 (T>TG). (f) chr2:144434823 (C>T). (g) mutation of chr2:144443640 (A>T). (h) mutation of chr2:144448665 (G>A). (i) chr2:144500762 (T>C). (j) mutation of chr2:144504145 (G>A).

a

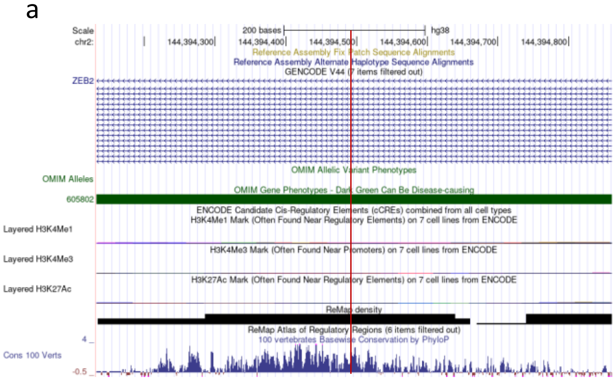

chr2:144394497 (A>G)

b

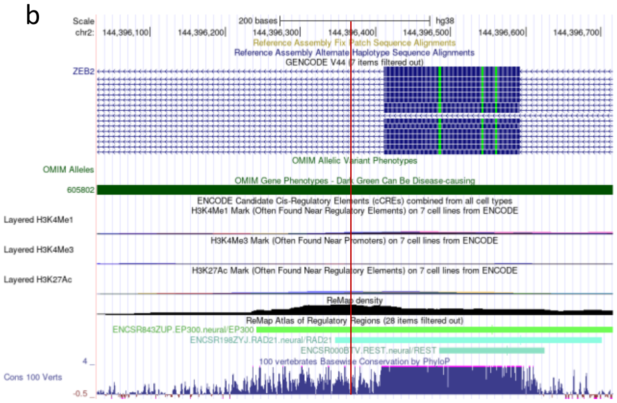

chr2:144396373 (G>C)

c

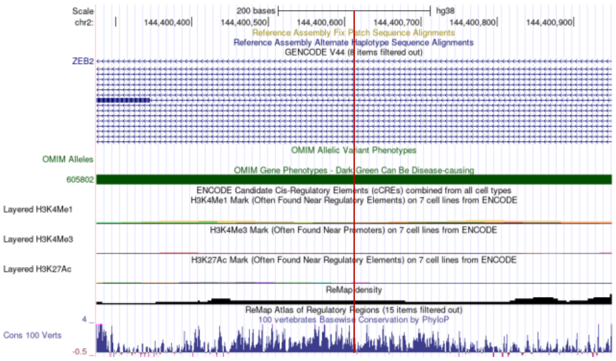

chr2:144400613 (A>G)

d

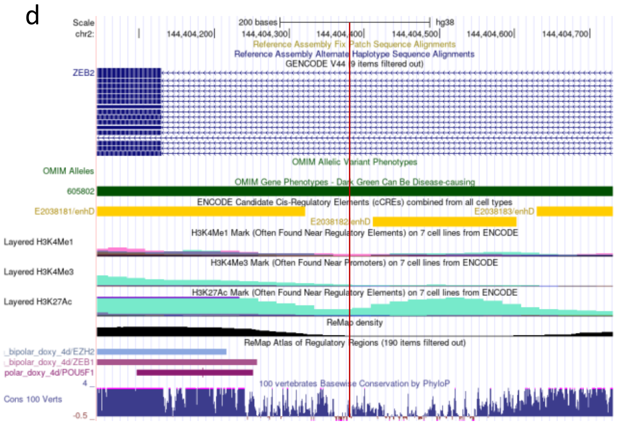

chr2:144404388 (T>C)

e

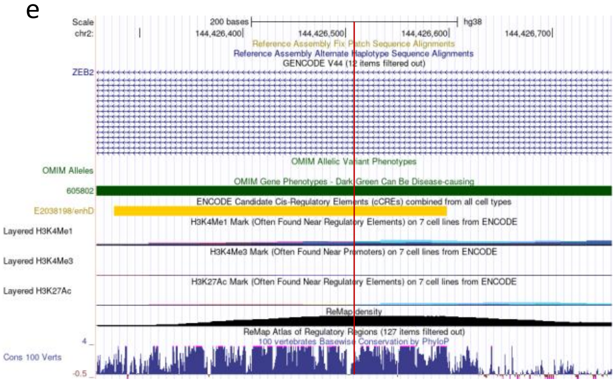

chr2:144426509 (T>TG)

f

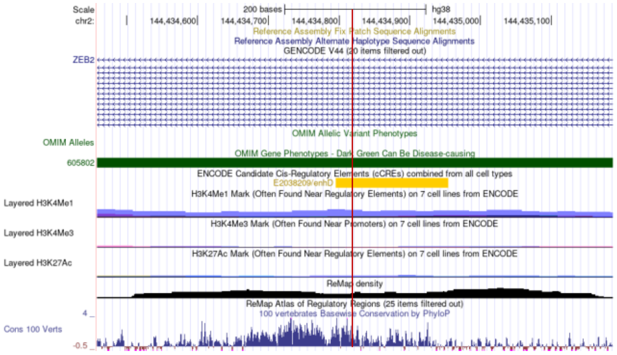

chr2:144434823 (C>T)

g

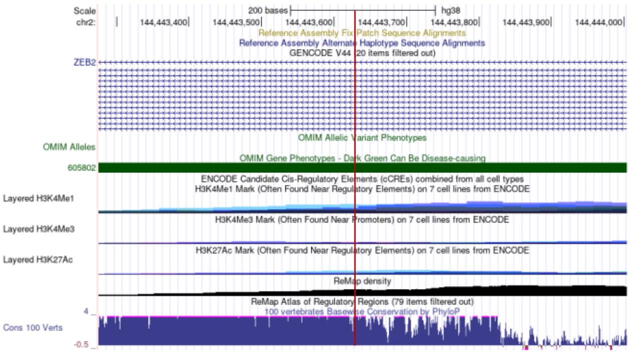

chr2:144443640 (A>T)

h

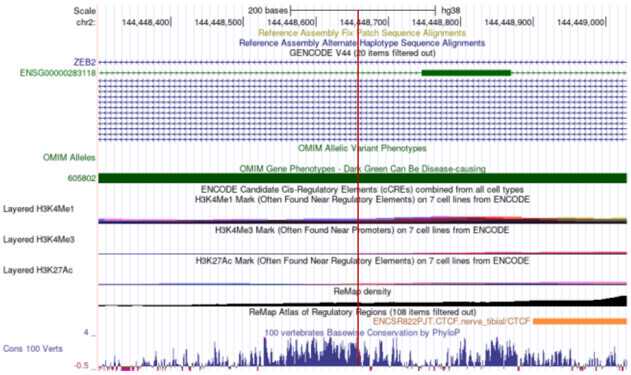

chr2:144448665 (G>A)

i

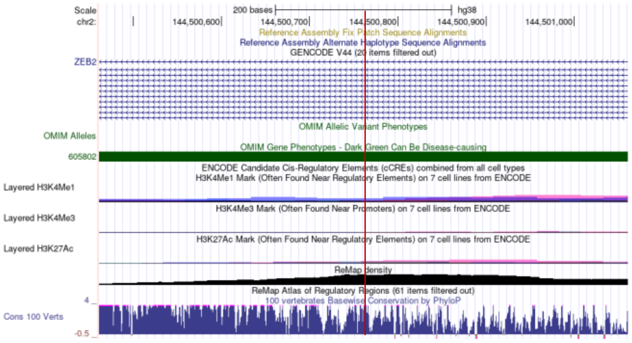

chr2:144500762 (T>C)

j

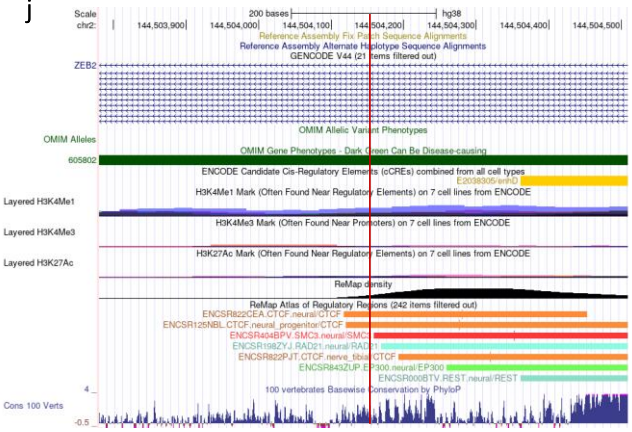

chr2:144504145 (G>A)

For *ZEB2* gene, three noncoding DNMs (2:144426509, T>TG; 2:144434823, C>T) identified from ASD cases are likely target cCREs with a distal enhancer-like signature. One noncoding DNM, located at position chr2:144404388 (T>C), showed higher enrichment of the H3K27Ac histone mark on HSMM Cells. Noncoding DNM located at position chr2:144504145 (G>A) exhibited higher enrichment of the H3K4Me1 histone mark on K562 Cells. Additionally, noncoding DNM of chr2: 144396373 (G>C) is situated 39 bp away from the exon, but not likely being splice-altering (SpliceAI score =0).

Fig S5. Mutation of the noncoding DNMs for *AGMO* in SPARK. (a) mutation of chr7:15490351 (T>C). (b) mutation of chr7:15502483 (G>A). (c) mutation of chr7:15542354 (T>C). (d) mutation of chr7:15556988 (T>C).

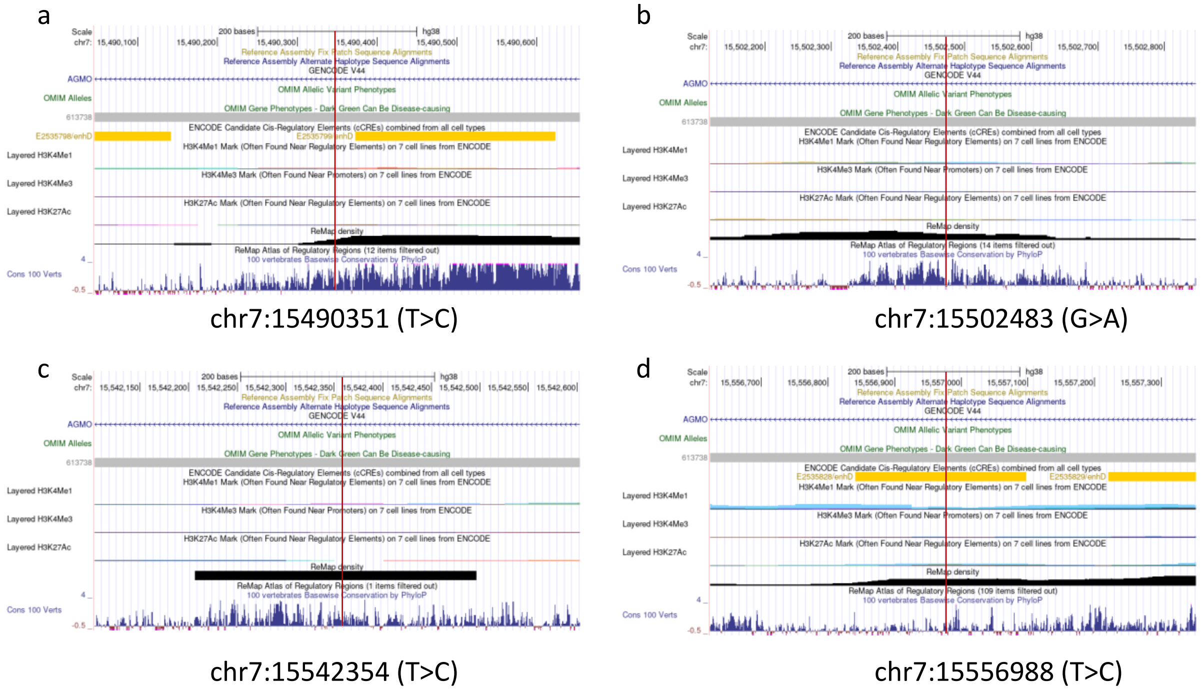

The *AGMO* gene harbored 4 noncoding DNMs in ASD cases. Noncoding DNM of chr7:15556988 (T>C) is likely candidate cCREs with a distal enhancer-like signature and slightly higher enrichment of the H3K4Me1 histone mark on HUVEC cells. Additionally, mutation of chr7:15502483 (G>A) shows slightly higher enrichment of the H3K4Me1 histone mark on HUVEC cells.
